# Analysis of sequence variations in the suppressor of cytokine signaling (*SOCS*)*-3 *gene in extremely obese children and adolescents

**DOI:** 10.1186/1471-2350-8-21

**Published:** 2007-04-19

**Authors:** Katja Hölter, Anne-Kathrin Wermter, André Scherag, Wolfgang Siegfried, Hanspeter Goldschmidt, Johannes Hebebrand, Anke Hinney

**Affiliations:** 1 Clinical Research Group, Department of Child and Adolescent Psychiatry, Philipps-University of Marburg, Germany; 2 Institute of Medical Biometry and Epidemiology, Philipps-University of Marburg, Germany; 3 Obesity Treatment Center Insula, Berchtesgaden, Germany; 4 Spessart Klinik, Bad Orb, Germany; 5 Department of Child and Adolescent Psychiatry, University of Duisburg-Essen, Essen, Germany

## Abstract

**Background:**

The suppressor of cytokine signaling (SOCS)-3 is a negative feedback regulator of cytokine signaling and also influences leptin signaling. We investigated association of variations in the coding sequence and promoter region of *SOCS3 *with extreme obesity in German children and adolescents.

**Methods:**

An initial screen for sequence variations in 181 extremely obese children and adolescents and 188 healthy underweight adults revealed two previously reported single nucleotide polymorphisms (SNPs) in the SOCS3 5' region: -1044 C>A (numbering refers to bases upstream of ATG in exon 2) within a predicted STAT3 binding element and -920 C>A (rs12953258, for numbering, see above).

**Results:**

We did not detect significant differences in allele or genotype frequencies for any of these SNPs between the analysed study groups (all nominal p > 0.2). In addition, we performed a pedigree transmission disequilibrium test (PDT) for the SNP -1044 C>A in families comprising 703 obese children and adolescents, 281 of their obese siblings and both biological parents. The PDT revealed no transmission disequilibrium (nominal p > 0.05).

**Conclusion:**

In conclusion, our data do not suggest evidence for a major role of the respective SNPs in *SOCS3 *in the pathogenesis of extreme obesity in our study groups.

## Background

Obesity is a growing health problem in both developed and developing countries and a major risk factor for cardiovascular disease. After the discovery of leptin, it was initially hoped that exogenous leptin therapy might induce satiety and weight loss in obese humans [1-3]. Inborn leptin deficiency was found to be extremely rare; only within a few consanguineous families homozygotes for leptin deficiency mutations have been detected [4,5]. In these cases, leptin supplementation normalized eating behaviour and reduced fat mass substantially [6-8]. However, the vast majority of obese individuals display elevated levels of circulating leptin as a consequence of their increased fat mass [9]. In these obese individuals, the weight loss achieved with recombinant leptin therapy had at the most been modest [10].

The fact, that obese individuals did not adequately respond to increased leptin levels with reduced food intake implicates that obesity is also influenced by leptin resistance [11]. One possible mechanism underlying leptin resistance is an inhibition of the intracellular leptin receptor signaling cascade [12]. Examples for an involvement of the inhibitor molecule suppressor of cytokine signalling three (SOCS3) in the regulation of leptin signaling include the following: (i) Exogenous administration of SOCS3 blocks leptin signaling in cultured cells, whereas leptin signaling is enhanced by knockdown of *Socs3 *and a mutation of the Socs3 binding site on the leptin receptor [13,14]. (ii) Expression of *Socs3 *is stimulated by leptin in cultured cells and in the hypothalamus, suggesting that high leptin levels attenuate leptin signaling by *Socs3 *expression [13-17]. (iii) Several rodent models of leptin resistant obesity exhibit increased *Socs3 *expression [13,17]. (iv) The demonstration of leanness and leptin sensitivity in mice haplo-insufficient for *Socs3 *and mice lacking *Socs3 *in the CNS suggests that Socs-3 limits leptin action *in vivo *[18,19].

SOCS3 belongs to the family of cytokine-inducible inhibitors of signaling including cytokine-inducible SH2 domain containing protein (CIS) and SOCS1–7 [20]. Members of this family are small proteins containing a N-terminal region of variable length, a central SH2 domain and a conserved C-terminal SOCS box [21]. The human *SOCS3 *gene maps to chromosome 17q25.3 and consists of two exons spanning 2,729 nucleotides [22]. The coding sequence in exon 2 (total of 2,401 nucleotides) comprises 678 nucleotides. The *SOCS3 *minimal promoter is 128 bp in length and consists of two adjacent STAT-binding elements (SBE), a G/C-rich element, and a putative TATA box [23]. These elements are highly conserved in both murine and rat *Socs3 *promoters [24,25].

Given the importance of leptin in body weight regulation, this study was undertaken to determine whether variations in the coding sequence (CDS) or promoter region of *SOCS3 *are associated with early onset obesity in a German population. We hypothesized that genetic variations, leading to an altered expression or function of *SOCS3 *could affect energy homeostasis through an influence on leptin signaling and thus predispose an individual towards the development of obesity.

## Methods

### Study subjects

The ascertainment strategy was previously described in detail [26]. All extremely obese children and adolescents had an age and gender specific BMI percentile of 97 or higher, the BMI of the underweight students was below the 15 th percentile, as previously determined in a representative German population sample [27]. Inclusion criteria for the underweight students additionally included: (a) absence of somatic disorders, and (b) consumption ≤ 10 cigarettes per day. Study group 1 comprised 181 extremely obese German children and adolescents who were recruited in hospitals specialised for treatment of young in-patients with extreme obesity. Study group 2 included 278 healthy underweight students who were recruited at the University of Marburg. The latter were reimbursed for their voluntary participation.

The family/trio sample included 703 families comprising a total of 984 extremely obese children and adolescents; their obese siblings and both biological parents.

Study groups 1, 2 and the family/trio sample were independent of each other. Table 1 shows the descriptive statistics of the genotyped individuals. All participants were Germans from a homogeneous population of European descent.

**Table 1:** Descriptive statistics (frequencies, means, standard deviations) for the investigated case-control and family sample.

sample^#^	n*	age [years]	BMI [kg/m^2^]	weight [kg]	height [cm]	waist hip ratio	lg [leptin]
underweight adults	278	24.98 (3.69)	18.24 (1.11)	59.13 (8.68)	176.66 (9.65)	0.79 (0.42)	0.23 (1.13)
female	142	24.61 (3.40)	17.55 (0.77)	51.98 (4.30)	169.90 (6.49)	0.79 (0.59)	1.21 (0.64)
male	136	25.37 (3.93)	18.96 (0.94)	65.63 (6.19)	183.72 (6.99)	0.80 (0.05)	-0.52 (0.80)
extremely obese children and adolescents	181	14.31 (2.40)	35.60 (6.12)	99.05 (24.93)	165.95 (11.78)	0.91 (0.10)	3.25 (0.60)
female	91	14.61 (2.41)	35.93 (6.59)	98.33 (23.51)	164.63 (9.94)	0.85 (0.08)	3.44 (0.48)
male	90	14.01 (2.37)	35.25 (5.64)	99.80 (26.42)	167.30 (13.33)	0.97 (0.09)	3.02 (0.65)
extremely obese offspring within 703 families	984	14.00 (3.69)	31.05 (5.98)	83.55 (24.26)	162.42 (14.10)	0.90 (0.09)	3.06 (0.75)
female offspring	543	14.01 (3.78)	31.27 (6.27)	81.58 (23.89)	159.89 (12.92)	0.86 (0.08)	3.19 (0.63)
male offspring	441	13.99 (3.59)	30.79 (5.60)	85.98 (24.52)	165.54 (14.85)	0.93 (0.08)	2.95 (0.85)

^# ^all subjects were recruited in Germany and all self-reported to be of European descent

* maximum number of observations for each variable

Written informed consent was given by all participants and, in the case of minors, their parents. This study was approved by the Ethics Committee of the University of Marburg and carried out in accordance with the Declaration of Helsinki.

### Molecular genetic methods

We designed three primer pairs to amplify the entire coding region (exon 2) of *SOCS3*. Primers were derived from a genomic sequence (GenBank accession no: NC_000017; 28) as follows: SOCS3-3d-F: 5' GTT CCG GGC ACT CAA CG 3' and SOCS3-3d-R: 5' CAG CTT GAG CAC GCA GTC 3': 497 bp SOCS3-3d amplicon. SOCS3-3-F: 5' AGT CTG GGA CCA AGA ACC TG 3' and SOCS3-3-R: 5' CTT GTG CTT GTG CCA TGT G 3': 499 bp SOCS3-3 amplicon. To amplify the minimal promoter region [23], we designed following primer pair: SOCS3-prom-F: 5' CTC GTC GCG CTT TGT CT 3' and SOCS3-prom-R: 5' GGG AGG GGA CCA GGA G 3': 344 bp SOCS3-prom amplicon. Polymerase chain reaction (PCR) was performed according to standard protocols. The SOCS3-3d amplicon was digested with *Gsu*I (Fermentas, resulting in fragments of 274 bp and 223 bp) and the SOCS3-3 amplicon was digested with *Nae*I (NEB, resulting in fragments of 279 bp and 220 bp). Single strand conformation polymorphism analysis (SSCP) was performed at ambient temperature and at 4°C, to increase the mutation detection sensitivity as described previously [29]. All PCR amplicons with SSCP patterns deviant from the wild-type pattern were re-sequenced as described previously [29]. To genotype -1044 C>A, PCR with subsequent diagnostic restriction length polymorphism analyses (RFLP) was performed: the 344 bp PCR amplicon SOCS3-prom was digested by *Mva *I (NEB; C-allele: 12, 157 and 175 bp, A-allele: 12 and 332 bp). To genotype rs12953258 (-920 C>A) we performed tetra-ARMS-PCR [30] with following primers: C-920A-o-F: 5' CCG CGC TCA GCC TTT CTC TGC TGC GA 3'; C-920A-o-R: 5' AGT CCA CAA AGG AGC CTT CGC GCG CG 3'; C-920A-i-F: 5' CCC GCA CGC AGC CAG CCG TCC 3'; C-920A-i-R: 5' AGC TGG GCC GGG CGG GCG ACT 3'. Sizes of the PCR products were 338 bp (outer primer pair), 210 bp (A-allele) and 170 bp (C-allele). All PCR products were visualized on ethidium bromide-stained 2.5% agarose gels. Allele sizes were determined with a molecular weight standard (123 bp ladder, Gibco BRL, Karlsruhe, Germany). Positive controls for the variant alleles were run on each gel. For validity of the genotypes, allele determinations were rated independently by at least two experienced individuals. Discrepancies were resolved unambiguously either by reaching consensus or by re-typing.

### Statistical analysis

Differences in genotype and allele frequencies between the independent study groups 1 and 2 were investigated using Fisher's exact test and in the case of genotypes the Cochran-Armitage Trend test. The pedigree transmission disequilibrium [PDT; 31] was performed for family-based analyses using UNPHASED [32] and estimates of genetic effects were derived according to Cordell and Clayton [33]. Power calculations were done with the software QUANTO Version 1.0 [34] for the situation of common variants. 180 case-control pairs were estimated to yield a power of 0.80 to detect a multiplicative genotype relative risk of 1.87 (α = 0.05; two-sided) assuming a minor allele frequency (MAF) of 0.1. For the family-based approach (700 trio families) the power estimate was 0.8 to detect a multiplicative genotype relative risk of 1.4 (α = 0.05; two-sided) again assuming a MAF of 0.1. Thus, the study was well powered to detect common disease predisposing variants of moderate effects. All genotype distributions were tested for Hardy Weinberg equilibrium (HWE) using PEDSTATS 0.6.4 [35] and no evidence for such deviations was detected (all p > 0.4). The level α for each test was 0.05 (two-sided) and due to the fact that we were not able to find evidence for a significant association, no correction for multiple testing was considered. If not mentioned otherwise, all reported p-values are nominal, two-sided and exact.

## Results

In the study groups comprising 181 extremely obese children and adolescents (study group 1) and 188 healthy underweight controls (study group 2) we detected two previously described SNPs: -1044 C>A; 36) within the promoter region and rs12953258 (-920 C>A; 37) in the 5'UTR (exon 1) of *SOCS3*.

We did not identify any sequence variants within the coding region. The linkage disequilibrium (r^2^) between the two markers (-1044 C>A) and rs12953258 (-920 C>A)) derived by FAMHAP (Version 16) [38] was <0.002 implying that they are largely independent. Evidence for differences in allele and genotype frequencies were not detected for the two SNPs between 181 extremely obese children and adolescents and 278 underweight subjects (all p > 0.2; table 2). Because of its potential functional consequences we additionally genotyped -1044 C>A in families comprising 703 extremely obese children and adolescents, 281 of their obese siblings and both biological parents. The pedigree transmission disequilibrium test (PDT) revealed 16 transmissions and 21 non-transmissions of the A-allele to the obese offspring (asymptotic p (SUM-PDT) = 0.3) which corresponds to an estimated genetic effect (odds ratio) of 0.76 with a 95% confidence interval (0.45–1.29) for heterozygous carriers compared to CC homozygous. The A-allele frequency estimated in the parents was 0.86% with a 95% confidence interval (0.55%–1.27%).

**Table 2:** Genotype distributions and estimates for the polymorphisms -1044 C>A and -920 A>C in the vicinity of *SOCS3 *for the case-control sample (percentages in parenthesis).

*SOCS3 *marker	underweight adults (n = 278)	extremely obese children and adolescents (n = 181)	estimated genotype odds ratio [exact 95% confidence interval]	estimated minor allele frequency in cases [Clopper- Pearson 95% confidence interval]
-920 C>A				
CC	235 (84.53%)	144 (79.56%)		
CA	40 (14.39%)	35 (19.34%)	1.43 [0.84–2.42]	10.77% [7.77%–14.43%]
AA	3 (1.08%)	2 (1.10%)	1.09 [0.09–9.61]	
-1044 C>A				
CC	270 (97.12%)	174 (96.13%)		
CA	8 (2.88%)	7 (3.87%)	1.36 [0.41–4.37]	1.93% [0.78%–3.94%]
AA	0 (0%)	0 (0%)	-	

## Discussion

Here we report an association study of *SOCS3 *sequence variations in body weight regulation in humans. We systematically screened the CDS and the minimal promoter of the human *SOCS3 *gene for sequence variations. In our study groups comprising 181 extremely obese children and adolescents and 188 healthy underweight adults we did not identify sequence variants within the CDS. Note that the screening sample size is sufficient to allow for the detection of common variants (e.g. when estimating a MAF of 0.05 a sample size of n = 180 produces a 95% confidence interval with a precision of +/- 0.02). In the *SOCS3 *minimal promoter region we identified the SNP -1044 C>A and in the 5'UTR (exon 1) the SNP rs12953258. Both SNPs were previously described with a minor allele frequency of 8% (rs12953258) and below 1% (-1044 C>A) in Danish individuals [36].

In a group of 360 population-based young healthy subjects the insulin sensitivity index was higher among homozygous carriers of the *A*-allele of rs12953258 than among heterozygous and wild-type subjects (*p *= 0.027, uncorrected; 36). However, an effect on BMI was not detected. A very recent large study did not find association between rs12953258 and two other SNPs (rs4969169 and rs8064821) in *SOCS3 *and obesity, insulin or lipid measures [39]. The study comprised 2,777 female twins of European descent, who were not ascertained for obesity or elevated body weight. This finding can be considered as representative of the UK female population. Both previous studies [36,39] were performed on population-based study groups; both did not find association of the rs12953258 alleles with obesity, which is in line with our findings. As our study relies on extremely obese individuals our results additionally to the previous results showed that the SNP is also not relevant for extreme obesity.

Our comparison of 181 extremely obese children and adolescents and 278 underweight adults revealed no evidence for an association for rs12953258. SNP -1044 C>A is located within the proximal SBE where the A-allele destroys the specific palindromic STAT binding motif TTCCAGGAA resulting in TT**A**CAGAA. Previous studies on the *SOCS3 *promoter indicated that particularly the proximal SBE is functionally relevant for promoter activity [24,25,40,41]. We hypothesized, that the -1044 C>A variant might result in the loss of a STAT3 binding site and thus influence the leptin dependent transcriptional activation of *SOCS3*. In our case-control association study we also compared allele and genotype frequencies of -1044 C>A. However, no evidence for an association with obesity was detected. Because of the potential functional role of this SNP and its infrequency we additionally performed PDT analyses in 703 families comprising 984 extremely obese children and adolescents and their obese siblings and both biological parents. The test revealed no evidence for transmission disequilibrium. However, the potential relevance of this variation in gene regulation warrants comparative functional studies.

We tried to answer the question if our finding negative because the selected SNPs had no effect on SOCS3 expression/function, or if variable SOCS3 expression/function has no effect on obesity. Gylvin et al. [36]*in silico *analysed that the -920 A allele deletes an activator protein 2 (AP2) transcription factor binding site. AP2 is involved in angiogenesis, tumour invasion/metastasis and chronic inflammation [42]. Additionally, the TRANSFAC database [43] also shows that the rs12953258 C-allele destroys a site bound by ZNF202, a transcriptional repressor, binding to elements found predominantly in genes that participate in lipid metabolism and therefore remains a variant of potential significance in SOCS3 action in lipid metabolism. Although there was no association between SOCS3 SNPs and obesity, this does not exclude a strong genetic determination of SOCS3 variability. The lack of association between the SOCS3 SNPs and obesity is probably because they are not in LD with a functional site.

Our study does not exclude the possibility that functional variants exist in the *SOCS3 *gene, which influence expression and function. On the basis of biological evidence, SOCS3 remains a strong candidate in this respect.

## Conclusion

Our results do not suggest a major role of the analysed SNPs in *SOCS3 *in early onset obesity. However, the relevance of this variation in gene regulation warrants functional studies. Since obesity is viewed as a polygenic disorder, products of numerous genes may be involved. To exclude moderate effects of the investigated sequence variations in body weight regulation large samples need to be assessed.

## Competing interests

The author(s) declare that they have no competing interests.

## Authors' contributions

KH carried out the molecular genetic studies, participated in the sequence alignment, made substantial contributions to conception, design and interpretation of data; she also drafted the manuscript. A-KW participated in the molecular genetic studies, and critically revised the manuscript. AS performed the statistical analysis and helped to draft the manuscript. WS and HG have made substantial contributions to the acquisition of data and revised the manuscript critically. JH and AH conceived the design, and participated in its design and coordination; helped to draft the manuscript and revised it critically. All authors read and approved the final manuscript.

## Pre-publication history

The pre-publication history for this paper can be accessed here:


